# The Influence of Computerized Tomography Imaging on Treatment of Periprosthetic Proximal Femur Fractures

**DOI:** 10.7759/cureus.38785

**Published:** 2023-05-09

**Authors:** Canon C Cornelius, Stephen Warner, Joel Thomas, David Rodriguez-Quintana

**Affiliations:** 1 Orthopedic Surgery, University of Texas Health Science Center at Houston, Houston, USA

**Keywords:** orthopaedic traumatology, orthopaedic adult reconstruction, revision arthroplasty, arthroplasty, proximal femur fracture

## Abstract

Introduction

Periprosthetic femur fractures (PPFF) are increasing in incidence and management of such injuries requires a specialized skill set combined with detailed knowledge of component design. To assist with planning, computed tomography (CT) can be used pre-operatively to give a surgeon more information. No study to date has shown the utility of obtaining preoperative CT. The goal of this study is to show that CT is a useful diagnostic adjunct and report any differences in how subspecialties such as orthopedic traumatologists and arthroplasty surgeons use it.

Methods

Seventeen PPFF cases met our inclusion criteria. They were shown to six faculty, three trauma and three arthroplasty surgeons. They viewed the plain radiographs and then CTs. After each they filled out the same questionnaire that included their assessment of diagnosis and proposed treatment plan both before and after access to CT imaging. Fleiss and Cohen kappa were used to compare inter- and intra-observer reliability.

Results

The interobserver kappa values (*k*) in diagnosis were 0.348 pre- and 0.371 post-CT, while trauma and arthroplasty were 0.328 to 0.260 and 0.821 to 0.881 respectively. The interobserver reliability in treatment was 0.336 pre- and 0.254 post-CT, while trauma and arthroplasty were 0.323 to 0.288 and 0.688 to 0.519. For intraobserver the average *k* for diagnosis and treatment were 0.818 and 0.671. Broken down by subspecialty they were 0.874 and 0.831 and 0.762 and 0.510 for trauma and arthroplasty. There were 11 diagnostic and 24 treatment changes.

Conclusion

CT provides diagnostic changes 10% and treatment changes 24% of the time. However, it does not lead to greater agreement among the surgeons on either. Arthroplasty uses CT more to guide both their treatment and the diagnosis, and they agree more than trauma surgeons. Most of the treatment changes come from adding or removing a plate, and the most common diagnostic change was shared by A to B1 and B2 to B3. This suggests fracture extension and bone stock are better evaluated by CT.

## Introduction

Periprosthetic femur fractures have been a concern since the first total hip arthroplasty (THA) was placed by Dr. Charnley [1]. The rates of both THAs and thus periprosthetic femur fractures are increasing [2,3]. Increase in periprosthetic fracture volume also increases the need for more knowledge of how to treat them and who should treat them. The treatment of these injuries requires knowledge of fracture care and reconstructive implant fixation options. Non-operative management, open reduction and internal fixation (ORIF), and ORIF in combination with revision arthroplasty are all possible management options. The final decision regarding treatment depends on multiple factors including implant stability, quality of bone stock, and fracture pattern among many others.

Historically the Vancouver classification has been used to classify and drive treatment [4]. This classification scheme is broken down into locations ie A, B, and C respectively being above, at, and below the implant. Furthermore, the B types are broken down into three separate categories based on implant stability and available bone stock, B1 being a stable implant, B2 and B3 being unstable, and B3 having inadequate bone stock. However, this classification system uses plain radiographs and there have been multiple studies that evaluated the system's inter- and intraobserver reliability [5,6]. Specifically, the misclassification of B2s as B1s can have devastating effects [7]. While plain radiographs should always be a first-line diagnostic tool, any way to obtain more information that informs the surgeon about treatment options and implant selection should be sought out. This highlights the main question of this study: Is there a role for pre-operative computed tomography (CT) in periprosthetic femur fractures? CT can show more detail than plain radiographs and these are commonly ordered in the acute evaluation of these periprosthetic fractures in our center.

The purpose of this study was to evaluate how CT scans affect the preoperative plans of surgeons, if that change is different among subspecialty groups, and reevaluate if CT can help predict the stability of an implant.

## Materials and methods

This was a retrospective review of patients with periprosthetic femur fractures treated by the arthroplasty faculty at our institution over a three-year period from 2017 to 2020. This study underwent IRB review and no approval was needed since it was retrospective in nature. There were 63 consecutive patients in the initial database. Two surgeons went through and separately graded them on the Vancouver scale. We included only Vancouver B types; this removed 15 cases. Then we viewed those that had computed tomography (CT). The CTs had to include the entire fracture. This brought the total number of patients down to 17; of these, we collected demographic data such as age and sex, preoperative radiographs, and CTs. The cases were completely deidentified and at this time a shareable presentation was made with anterior posterior (AP) and lateral radiographs and axial and coronal CTs.

**Figure 1:**
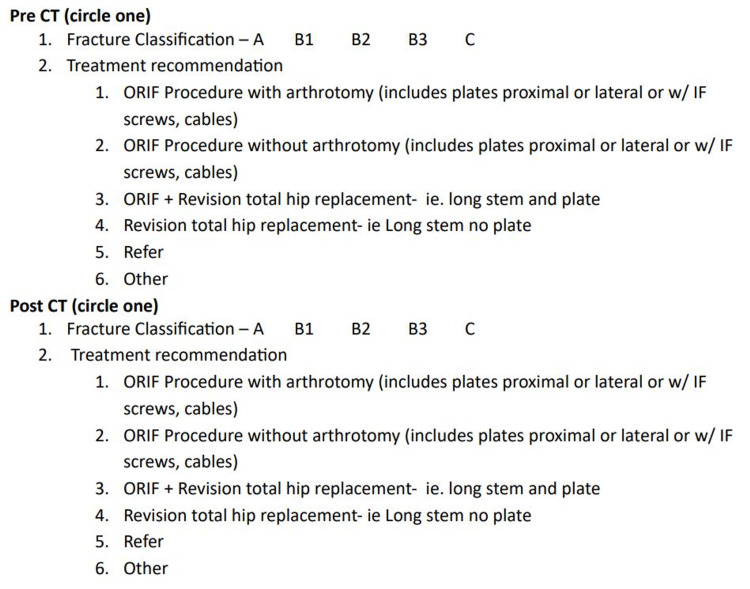
Questionnaire shown to attending physicians. ORIF: open reduction and internal fixation

Six attending orthopedic surgeons, three arthroplasty and three trauma fellowship-trained, viewed the cases. They view them in a similar way to how they would in real practice with plain radiographs first then followed by the CTs. Immediately following the radiographs-only section they classified the fracture according to the Vancouver classification and answered how they would treat it in a multiple-choice format. We included five different operative options: Non-operative, ORIF no arthrotomy, ORIF with arthrotomy, revision with plate, and revision without a plate. Then they reviewed the CT and answered the exact same questions (Figure 1). The goal of the questionnaire was to distinguish responses of different attending physicians and how CT was affecting their diagnosis and plan. 

Fleiss and Cohen kappa scores were used respectively for the interobserver and intraobserver reliability. To interpret the data the cutoffs described by Landis and Koch criteria were used (Table 1) [8].

**Table 1:** Kappa statistic explanations

Kappa Statistic	Strength of agreement
<0.00	Poor
0.00-0.20	Slight
0.21-0.40	Fair
0.41-0.60	Moderate
0.61-0.80	Substantial
0.81-1.00	Almost perfect

Assessment for stem stability, the gold standard being intraoperative assessment with arthrotomy, was calculated by simple percentage correct among the observers and overall complete agreement. Numbers needed to treat for both diagnostic and treatment purposes were calculated in the standard manner. Statistical Package for Social Sciences (SPSS; IBM Corp., Armonk, NY, USA) was used to perform data analysis. 

## Results

The mean age of the population was 79 with a range from 59 to 96 and 10 out of 17 were female (59%). Based on the intraoperative assessment six out of the 17 cases had stable implants in the OR and were treated as B1s in data analysis whereas the remaining 11 were treated as B2/3s. There were a total of three reoperations on these cases one for infection, one for implant failure, and one for fracture distal to the stem. There were no obvious cases of incorrectly treated stem stability.

The overall interobserver reliability kappa values in pre-CT diagnosis were 0.348 (95% CI 0.346-0.351), and 0.371 (95% CI 0.368-0.374) with the post-CT results. Specialty-specific interobserver reliability in diagnosis was as follows: Arthroplasty pre- and post-CT were 0.821 and 0.881 respectively and trauma’s were 0.328 and 0.260.

The overall interobserver reliability kappa values in pre-CT treatment were 0.336 (95% CI 0.334-0.336) and 0.254 (95% CI 0.252-0.256) with the post-CT results. Specialty-specific interobserver reliability in diagnosis was as follows: Arthroplasty pre- and post-CT were 0.688 and 0.519 respectively and trauma’s were 0.323 and 0.288. Within the treatment categories the biggest difference between trauma and arthroplasty surgeons was their desire to perform an arthrotomy with arthroplasty-trained attendings choosing to do so 27 times (27%) and trauma surgeons only suggesting it four times (4%).

Intraobserver reliability was also assessed individually. Overall the average kappa values for diagnosis and treatment were 0.818 and 0.671 (Table 2).

**Table 2:** Individual and group kappa scores

	Diagnosis Kappa	Treatmemt Kappa
Arthroplasty 1	0.823	0.390
Arthroplasty 2	0.636	0.596
Arthroplasty 3	0.827	0.544
Trauma 1	0.854	0.754
Trauma 2	0.871	0.901
Trauma 3	0.897	0.838
Trauma-all	0.874	0.831
Arthroplasty-all	0.762	0.510
Overall	0.818	0.671

However, the trauma intra-observer reliability for diagnosis and treatment were 0.874 and 0.831, whereas the arthroplasty intra-observer reliability for diagnosis and treatment were 0.762 and 0.510.

There were 11 total diagnostic changes, with three (27%) of them coming from the trauma surgeons. The most common is shared by A to B1 and B2 to B3 with four each. Treatment changes in total were 24, with five (21%) of them coming from the trauma staff. The most common two changes were revision plus plate to revision alone and the reverse making up seven and six respectively for a total of 54% of overall changes.

When compared to intraoperative records the overall correct stability was the same pre- and post-CT at 80%. The absolute agreement instability was nine of 17 (53%) for the pre-CT analysis and eight of 17 (47%) for the post-CT. Of note, the absolute agreement for the arthroplasty in the pre-CT stability was 100%.

## Discussion

A study by Rupp et al. recently showed that the CT did not help improve preoperative evaluation of implant stability with the evaluation of implant stability [9]. This study did not assess the classification of the fractures or ask any treatment-related questions. CTs have been shown to affect treatment in other aspects of fracture care [10]. However, CTs also cost money and increase radiation exposure to the patient [11]. Implant stability, reconstruction options, and frailty of patients affected make periprosthetic femur fractures some of the more difficult injuries to deal with in orthopedic trauma and joint reconstruction. The first step in management of these complex injuries is deciding whether the implant has remained stable around the fracture. CT scan has shown some value in decision-making in multiple orthopedic injuries [10].

There was moderate agreement between all subjects in interobserver reliability between both the pre-CT and post-CT diagnoses. This lack of improvement suggests that the CT does not help surgeons agree on the diagnosis. However, when broken down into subspecialties the change in agreement is still not apparent but arthroplasty-trained surgeons appear to agree much more on the diagnosis almost perfect agreement on diagnosis both pre- and post-CT.

The pre- and post-CT treatment agreement was similar with fair agreement in both categories. However, the trend of the arthroplasty surgeons agreeing more with each other appears to be continued, at interobserver kappas in the moderate and substantial. The interesting component of that is the lower agreement was in the post-CT again suggesting that maybe CT doesn’t only not help with agreement but may make it worse. Another interesting observation here is the difference in belief in arthrotomy. These results suggest that if the trauma surgeons think the implant is stable preoperatively they will plan for ORIF without confirmation whereas the arthroplasty surgeons choose to confirm implant stability with an arthrotomy. CT did not appear to affect the decision to perform an arthrotomy in either subspecialty.

**Figure 2:**
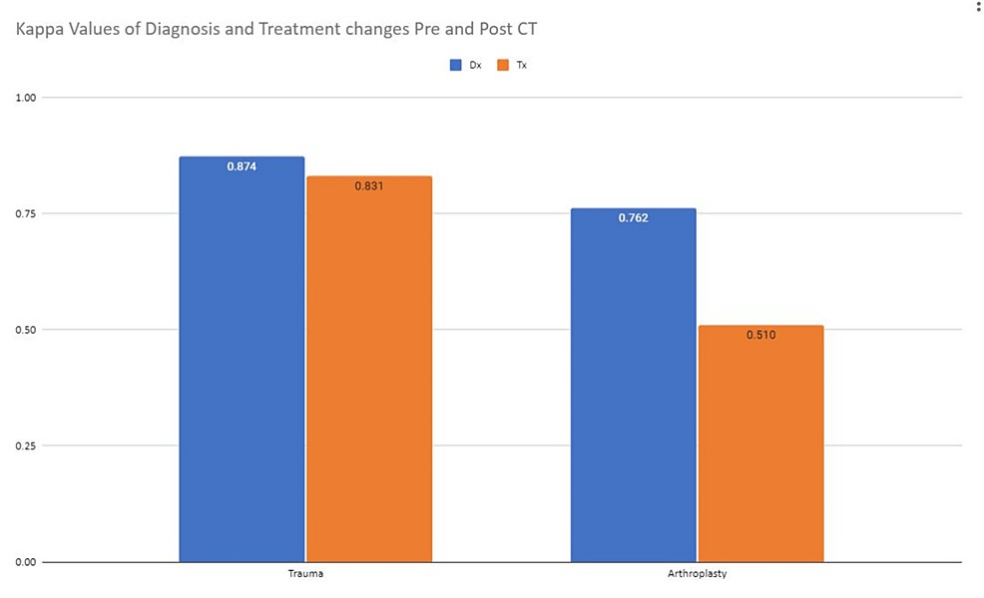
Kappa values pre- and post-CT by subspecialty Dx-Diagnosis, Tx-Treatment

The intra-observer kappa scores for the surgeons were averaged but can be seen individually in Table 2. The average kappas for all participants were 0.818 and 0.671 for diagnosis and treatment respectively (Figure 2).

This leads to “almost perfect agreement” for diagnosis suggesting that the diagnosis is not often changed with CT but that treatment is changed more often. The difference is seen even more so in the subspecialty breakdown. Trauma rarely changes diagnosis or treatment however it appears that arthroplasty uses the information from the CT to guide both their diagnosis and especially their treatment. All of this suggests that the use of CT can drive treatment more than previously published [9].

The knowledge of fracture care and implant type is imperative. Stability of stem and quality of the bone stock are two important factors. The overall correct prediction of stem stability was 80% pre- and post-CT, with very few changes in stability prediction based on CT. There was a trend towards the arthroplasty trained attendings being better at predicting stem stability with them being right 88% of the time whereas the trauma trained attendings correctly predicted stem stability 74% of the time. The two most common diagnostic changes were between A and B1 and B2 and B3. While not powered to prove significance this suggested that the CT may reveal non-displaced fracture lines about the stem not evident on plain radiographs as well as provide a better assessment of bone stock. The changes in treatment reflect this trend as well with the most common change being from revision with a plate to revision without a plate and vice versa. This combined with one of the most common diagnosis changes being B2-B3 leads one to believe that surgeons could be relying heavily on CT to assess bone stock.

There are several limitations to this study. The first being that there is no gold standard to predict what the correct diagnosis or treatment is. This paper is more of a comment on the rate and how surgeons may change their diagnosis and treatments, not necessarily on if they are correct. Another is the number of cases and surgeons participating. However, the number of periprosthetic femur fractures with full radiographic and CT imaging is a difficult thing to find in a retrospective manner. The time commitment to look at all the imaging, especially the critical evaluation of the CT makes it somewhat difficult to find additional participants. Future directions of this study include a round table discussion with the participants to discuss what they found helpful in the CT imaging and see if there are predictors of plains radiographic changes to allow for a formation of a protocol for when to obtain CT in periprosthetic femur fractures.

## Conclusions

In summary, CT is a useful tool and likely worth the additional cost and radiation. Our results suggest that due to the changes in diagnosis and treatment seen in this study 10 CTs are needed to change the diagnosis and that between four and five CTs are needed to change treatment. It is the belief of the authors that those changes are significant. This is mostly due to the catastrophic failures that can occur when fracture characteristics are underappreciated.
